# Contributions to Operative Surgery, and Surgical Pathology

**Published:** 1860-04

**Authors:** 


*Contributions to Operative Surgery, and Surgical Pathology, by Prof. J. M. Carnochan, M. D.* Part three. 4to. 1860. Lindsay & Blakiston, Publishers, Philadelphia.

In announcing Part Three of this valuable work, we again recommend it to our readers' attention. It is, in fact, indispensable to all who desire to become acquainted with some of the most remarkable and important achievements of modern surgery. The part before us, among other articles, contains some remarks "on the Restoration of the Entire Upper Lip, with Cases." This will be found particularly interesting and instructive. It relates to an operation lately performed for the first time in this country, by Professor Carnochan, with complete success.

